# The Development of a Short Chinese Version of the State-Trait Anxiety Inventory

**DOI:** 10.3389/fpsyt.2022.854547

**Published:** 2022-05-09

**Authors:** Qingyun Du, Haowen Liu, Chengjiao Yang, Xiaoyu Chen, Xiaoyuan Zhang

**Affiliations:** ^1^Department of Psychology, School of Public Health, Southern Medical University, Guangzhou, China; ^2^Department of Psychiatry, Zhujiang Hospital, Southern Medical University, Guangzhou, China

**Keywords:** STAI, short version, patient, Chinese, STIA-S

## Abstract

**Background:**

Few studies on anxiety in China have used the full version of the Spielberger State-Trait Anxiety Inventory (STAI) due to its length, despite its numerous advantages. The goal of this study was to develop a short Chinese version of the STAI and examine its reliability and validity among the general Chinese population and psychiatric patients diagnosed with anxiety.

**Method:**

A total of 1,142 participants were invited to test the short Chinese version of the STAI; item analysis, validity testing and internal consistency reliability analysis were performed. Subsequently, 40 participants were enrolled to assess retest reliability 1 week later. Finally, 330 participants, including 33 psychiatric patients with anxiety, were used to assess the criterion and empirical validity. The Self-Rating Anxiety Scale (SAS) and Satisfaction with Life Scale (SWLS) were used as criteria, and receiver operating characteristic (ROC) analysis was conducted to evaluate the discrimination of the short version of the STAI between the groups with and without anxiety disorders.

**Result:**

The short Chinese version of the STAI contains six items for each subscale (STAI-S-6 and STAI-T-6). The Pearson correlation coefficients between the two shortened Chinese versions of the STAI and the full-length STAI were 0.970 and 0.962, the Cronbach’s α coefficients were 0.871 and 0.842, and the retest reliability values were 0.728 and 0.813 (*p* < 0.001). Confirmatory factor analysis showed that the 2-factor model achieved an adequate model fit: for the STAI-S-6, CFI = 0.986, TLI = 0.974, and RMSEA = 0.075, and for the STAI-T-6, CFI = 0.994, TLI = 0.988, and RMSEA = 0.052. The short Chinese version of the STAI had a significant positive correlation with the SAS score (*r* = 0.289, 0.313; *p* < 0.001) and a negative correlation with the SWLS score (*r* = −0.476, 0.554; *p* < 0.001). A significant difference in the level of anxiety was found between participants with and without anxiety disorders. The diagnostic accuracy of the STAI-S-6 and STAI-T-6 for major anxiety disorder was acceptable, with areas under the ROC curves of 0.798 and 0.745, respectively.

**Conclusion:**

The short Chinese version of the STAI demonstrates sound psychometric properties and is applicable in evaluating the level of anxiety in Chinese populations.

## Introduction

The State-Trait Anxiety Inventory (STAI) developed by Spielberger et al. (1) is one of the most commonly used self-report measures of anxiety in research and clinical settings across cultures (2). As of 2020, more than 388 published studies reported the association of the STAI trait scale (STAI-T) with depression (3). This instrument provides an operational measure of two components of anxiety, state and trait anxiety, which refer to a transitory emotional state characterized by subjective feelings or tension that may vary in intensity over time and to a relatively stable disposition to respond to stress with anxiety and a tendency to perceive a wider range of situations as threatening, respectively (4). Based on these two types of anxiety, the STAI consists of two scales measuring state anxiety (STAI-S) and trait anxiety (STAI-T). The STAI-S scale consists of 20 items: half of these items are positive items (anxiety-absent), and the other half are negative items (anxiety-present). The original STAI-T scale consisted of seven items that were positively worded (anxiety-absent), and the other 13 items were negatively worded (anxiety-present). The original STAI, termed STAI-X, was revised in 1983 (5). This new version, known as the STAI-Y, replaced some of the original STAI items related to depression to improve its specificity. The STAI-Y also improved the structure of the STAI-T scale by achieving a better factor structure balance between anxiety-present and anxiety-absent items. The STAI-Y is used in many clinical areas, such as alcohol use disorder (6), providing normative data on anxiety (7, 8) and evaluation of treatment options (9). Although the STAI-Y appears to have better psychometric properties than the STAI-X, both instruments appear to be comparable for anxiety assessments, as the correlation between them ranges from 0.96 to 0.98 (5).

The development of a shortened version of a full-length scale is an important issue in research and clinical settings to reduce measurement time but not reduce the measurement value of the original X scale. Marteau and Bekker (10) developed the first short-form of the STAI-S scale from the STAI-Y and suggested that the STAI-S scale can be reduced to an abbreviated form. They selected three anxiety-present items and three anxiety-absent items to maintain the balance between the anxiety-present and anxiety-absent dimensions of the STAI-S scale. The scores of this 6-item short-form STAI-S scale are highly correlated with the full 20-item scale and have been widely used in basic research and clinical practice (11–13).

Many countries have also developed short versions of the STAI scale, for example, a 6-item version from the Netherlands (14), 5-item version from Japan (15), 8-item version from France (16) and 6-item version from Brazil (17). In addition to the study in Brazil, the other researchers did not try to test whether the short versions of the STAI scales could be used to examine two aspects, anxiety-present and anxiety-absent, which existed when Spielberg et al. developed and revised the original full length of the STAI scales.

The purpose of the present study was to develop a short form of these two STAI scales that followed the original structures of the STAI scale that contains anxiety-present and anxiety-absent factors in each of the S/T scales with acceptable psychometric properties in a Chinese population and to conduct a comprehensive evaluation to fill the gap in intervention methods. Based on the Chinese epidemiological investigation of anxiety disorders (18), high anxiety diagnosis rates have been reported in adults undergoing dental procedures (19) (26.6%), elderly persons (20) (7.86%) and guardians of vaccinated children (21) (7.1%). However, most patients with anxiety disorders are not identified, partially because of the variable clinical manifestations of anxiety disorders and because clinicians lack confidence in making an accurate diagnosis. Therefore, a tool for evaluating anxiety with good reliability, validity, specificity, and sensitivity is important. The short STAI scale developed in this study should provide good assistance for clinical practice. Although similar efforts have been attempted, Tian et al. (22) translated the SSTAI (Short State Anxiety Inventory, SSAI) short-form scale developed by Marteau and Bekker, and its reliability and validity were also tested (Cronbach’s α = 0.81, KMO = 0.79). However, it was directly translated from the reduced STAI-S scale, and the items were selected based on the data from foreign samples. No studies have attempted to develop a short version of the STAI-S and STAI-T scales by selecting items based on samples in China, where the need for an efficient anxiety evaluation method is also urgently needed due to the large number of patients treated in outpatient departments, either psychiatric clinics or general hospitals (23, 24).

We suggest a reliable, short Chinese version of the STAI scale to help practitioners evaluate participants’ anxiety status in a short time to achieve the goal of our study in general. We conducted our study in two steps. In the first step, we selected the items with the best item-total correlation representing the anxiety-present and anxiety-absent factors of the STAI-S/T scales. We also performed confirmatory factor analysis to test whether the two-factor model fit the data of the Chinese population (25). Meanwhile, we evaluated the retest reliability of short-form STAI scales.

In the second step, we conducted tests to analyze the sensitivity, specificity and calibration validity of short-form STAI scales.

## Materials and Methods

### Participants

#### We Carefully Chose Participants for Each of the Steps Described Below to Choose the Most Representative Items for the Short Form and Test Its Sensitivity, Validity, and Reliability

##### Step 1

We selected optimal items for the short version scale using the method described below.

A group of 1,142 participants (527 males and 615 females, mostly aged 18–40 years) from two samples participated in this part of the evaluation. One sample included 571 full-time students, and the other included 571 employed participants. Data were collected with the Chinese non-profit online questionnaire platform “WJX” in December 2020. The participants resided in more than 30 provinces and reported more than 10 occupations, and most of them had a monthly income between 4,000 and 10,000 RMB. A total of 1,188 questionnaires were sent out, and 1,142 were effectively returned, with a return rate of 96.12%.

Test-retest reliability was evaluated using the method described below.

In May 2021, 42 participants (five males and 35 females aged 18–50 years) were contacted through WeChat and other social media platforms; we invited participants to select items for the short version of the STAI. A total of 33 full-time students from the same university and seven employed people were recruited. All of them provided consent for the follow-up procedure and contact methods. Two invalid data points were removed because two participants failed to complete the test within the specified time interval.

##### Step 2

Clinical value was evaluated using the method described below.

A group of 330 participants from two different samples participated in this step. The first sample included 297 participants without anxiety symptoms who were recruited by student assistants from Southern Medical University in April 2021. The return rate was 95.42%. The second sample included 33 participants with anxiety who visited the outpatient clinic of Zhujiang Hospital. A psychiatric diagnosis was made according to the Diagnostic and Statistical Manual of Mental Disorders, fourth edition (DSM-IV). The classification of “anxiety” for inclusion in the study was based on the criteria for generalized anxiety disorder and included those with clinically significant symptoms combined with social dysfunction but who did not meet the full criteria for a specific disorder (which is fairly common in China). The psychiatrist who participated in the study had experience working with patients with mental disorders for over 10 years and specialized in the diagnosis of mood disorder patients. The return rate was 97.42%.

All the demographic data of the participants in this study are listed in Table 1 below.

**TABLE 1:** Demographic data of the participants in the present study.

Sample	Sex (male/female)	Age range	Monthly income
**Step 1: To select optional items for the short version of the scales**			
Full-time students (*n* = 571)	214/357	Under 18 years (*n* = 33)	Under 2 k (*n* = 468)
		18–25 years (*n* = 512)	2 k–4 k (*n* = 77)
		26–30 years (*n* = 23)	4 k–6 k (*n* = 13)
		31–40 years (*n* = 3)	6 k–8 k (*n* = 1)
			8 k–10 k (*n* = 3)
			Above 10 k (*n* = 9)
Occupational workers (*n* = 571)	313/258	Under 18 years (*n* = 2)	Under 2 k (*n* = 39)
		18–25 years (*n* = 135)	2 k–4 k (*n* = 73)
		26–30 years (*n* = 185)	4 k–6 k (*n* = 119)
		31–40 years (*n* = 173)	6 k–8 k (*n* = 84)
		41–50 years (*n* = 59)	8 k–10 k (*n* = 118)
		51–60 years (*n* = 16)	Above 10 k (*n* = 138)
		Over 60 years (*n* = 1)	
**Step 1: To evaluate test-retest reliability**			
Full-time students (*n* = 33)	Apr-29	18–25 years (*n* = 33)	Under 2 k (*n* = 25)
			2 k–4 k (*n* = 8)
Occupational workers (*n* = 7)	01-Jun	18–25 years (*n* = 4)	Under 2 k (*n* = 1)
		26–30 years (*n* = 1)	2 k–4 k (*n* = 1)
		41–50 years (*n* = 2)	4 k–6 k (*n* = 3)
			6 k–8 k (*n* = 1)
			8 k–10 k (*n* = 1)
**Step 2: Evaluation of the criterion and empirical validity and clinical value**			
Non-anxiety Disorder Participants (*n* = 297)		Under 18 years (*n* = 5)	Under 2 k (*n* = 209)
	116/181	18–25 years (*n* = 253)	2 k–4 k (*n* = 44)
		26–30 years (*n* = 11)	4 k–6 k (*n* = 25)
		31–40 years (*n* = 12)	6 k–8 k (*n* = 5)
		41–50 years (*n* = 13)	8 k–10 k (*n* = 7)
		51–60 years (*n* = 3)	Above 10 k (*n* = 7)
		Under 18 years (*n* = 6)	Under 2 k (*n* = 3)
Anxiety Disorder Patients (*n* = 33)	Dec-21	18–25 years (*n* = 10)	2 k–4 k (*n* = 3)
		26–30 years (*n* = 1)	4 k–6 k (*n* = 8)
		31–40 years (*n* = 5)	6 k–8 k (*n* = 3)
		41–50 years (*n* = 9)	8 k–10 k (*n* = 8)
		51–60 years (*n* = 1)	Above 10 k (*n* = 7)
		Over 60 (*n* = 1)	

### Instrument

#### State-Trait Anxiety Inventory

The STAI questionnaire was translated and introduced in China in 1988. A study published in 1995 (26) was conducted to determine the norm in China. Items 1–40 that we used in our study were the same as the 1995 translated Chinese version of the STAI-Y. Four items from the 1995 Chinese version of the STAI were again translated into Chinese in this study because they were no longer suitable for the current situation and could lead to misunderstanding. The final testing scale contained a 20-item state scale and a 20-item trait scale that were the same as the Chinese version published in 1995, as well as four newly translated items. The four new items were first translated into Chinese by a psychologist who has international experience and is familiar with emotional problems and then backtranslated to English by two other translators who majored in English and had not seen the original items. The back translation was compared with the original English items by one of the authors to consider the linguistic and semantic equivalence between translations. All of the subjects participating in the pilot study were able to clearly understand the four new items, but they were eliminated after Step 1, as we selected optional items. The STAI scale requires the subjects to describe self-evaluated feelings toward 40 items with a range of 1–4 points possible for each item. Positive items should be scored oppositely. The sum of the scores for the state and trait anxiety scales was calculated separately, and higher scores indicated more severe anxiety. In this study, the Cronbach α of the STAI-S was 0.950, and the Cronbach α of the STAI-T was 0.926.

#### The Self-Rating Anxiety Scale

The Self-Rating Anxiety Scale (SAS), developed by Zung (27), was adopted to evaluate the participants’ anxiety states; its split half correlation coefficient was 0.71. The Chinese version was translated by Wang (28), and its criterion validity compared with the Hamilton Anxiety Rating Scale (HAM-A) was 0.365, which indicated that the validity of the SAS was quite high. The SAS includes 20 items, each with a score of 1–4 points. The sum of the scores of all items is the raw score, which was used to calculate the standard score using the following equation: the standard score equals the raw score multiplied by 1.25. Higher scores suggest a greater severity of depression or anxiety. In this study, Cronbach’s α was 0.862.

#### The Satisfaction With Life Scale

The satisfaction with life scale (SWLS), concerning subjective well-being, was developed by Diener et al. (29). The test-retest reliability was 0.82, and Cronbach’s α was 0.87. We used the Chinese version of the SWLS, which was translated and tested by Xiong (χ^2^ = 33.56, χ^2^/df = 6.71, GFI = 0.97, CFI = 0.96, RMSEA = 0.071, Cronbach’s α = 0.78) (30). It is assessed by measuring cognitive self-judgment about satisfaction with one’s life. It consists of five items presented on a 7-point Likert scale with ratings ranging from “Strongly Disagree” that score 1 point to “Strongly Agree” that score 7 points. High scores on the SWLS indicate greater satisfaction with one’s life. In this study, Cronbach’s α was 0.881.

### Procedure

#### Step 1

For the aim of selecting items for the short version, a quantitative survey considering the STAI was carried out. Participants were recruited using the internet. Questionnaires were completed with no time limit. None of the participants showed difficulty in understanding either the instructions or any of the items.

For the aim of assessing the test-retest reliability, forty-two participants recruited through the internet completed the revised short Chinese version of the STAI, and 1 week later, they were asked to complete the same short Chinese version of the STAI again. Two of them failed to complete the questionnaires at the time allotted, and others had no questions about the scales.

#### Step 2

For the aim of evaluating the empirical and criterion validity and the areas under the ROC curves, a quantitative survey using the STAI-S-6, STAI-T-6, SAS, and SWLS was carried out. Two hundred ninety-seven participants without anxiety disorder or any other mental problems and 33 participants diagnosed with only anxiety disorder were invited through the internet and by a psychiatrist. The individuals without anxiety disorder completed the scales online. The individuals with anxiety disorder completed paper forms of scales, and the results were subsequently input into computers by researchers. The participants had no questions about the scales, and all the scales were completed with no time limit.

**FIGURE 1:**
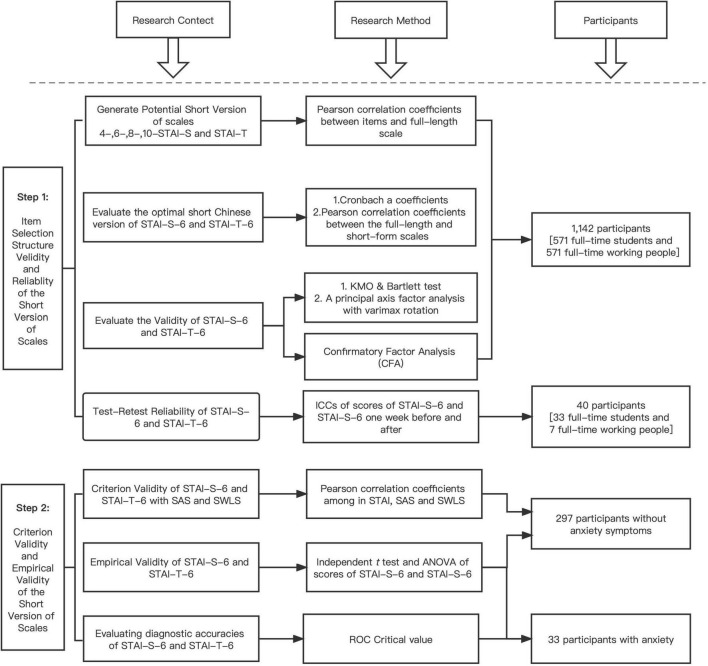
Prosess map.

After completing the scales, participants from both steps (Step 1 and Step 2) had an opportunity to receive a small amount of money at random through the WeChat red envelope (process see Figure 1).

### Statistical Analysis

For all analyses, we used response time and trap questions in the questionnaire (e.g., where does the sun rise) to eliminate invalid questionnaires and retain only effective questionnaires for data analysis, which effectively improved the effectiveness of the research to a certain extent. STAI, SAS, and SWLS sum scores were calculated as integers. Statistical analyses were performed in the order described below using IBM SPSS Statistics, version 26.

#### Step 1

The aim of Step 1 was to choose the optimal short forms of the STAI-S and STAI-T scales. The item selection procedure was used based on the statistical methodology reported by Marteau and Bekker (10). According to this procedure, items from the STAI-S and STAI-T scales were ranked according to their corrected item-total correlation scores. Based on the correlation, an equal number of anxiety-absent (positive) and anxiety-present (negative) items were chosen to generate 10-, 8-, 6-, and 4-item forms of the STAI-S and STAI-T scales. Then, the internal consistency of each of the four short-form versions was assessed by calculating their respective Cronbach’s α coefficients. Acceptable Cronbach’s α coefficients are usually greater than 0.7 (31). The similarity of the four short-form and full-length scales was evaluated by calculating Pearson correlation coefficients. A correlation value greater than 0.9 is generally accepted as a good indicator of proportionality between scales (32). In the evaluation process, the internal consistency of the four short versions of the STAI-S and STAI-T scales were evaluated. We also used the critical value (CR) of each item for which we chose to test the discrimination, and if the CR reached a significant level, it indicated that the item effectively identified the characteristics of the subjects and should be retained (33). In addition, confirmatory factor analysis was applied to examine the structure of the short version.

Intraclass correlation coefficients (ICCs) were calculated to evaluate the test-retest reliability of the STAI-S-6 and STAI-T-6 scales. The scale scores were recorded for a group of participants who were invited to answer the same questionnaire twice, with a 1-week interval. The higher the ICC is, the more stable the scale. ICC values greater than 0.75 are considered to indicate good reliability, whereas values less than 0.75 represent poor to moderate reliability (34).

#### Step 2

The aim of Step 2 was to prove the criterion validity of the STAI-S-6 and STAI-T-6 scales and to effectively distinguish individuals with anxiety from individuals without anxiety. The correlations of the STAI-S-6 with the SAS and SWLS scales were determined by calculating Pearson’s correlation coefficients for the scores of 297 participants without anxiety. The STAI-T-6 scale was processed similarly.

We performed receiver operating characteristic (ROC) curve analysis using data from 33 participants with anxiety and 297 participants without anxiety to assess the diagnostic accuracy of the STAI-S-6 and STAI-T-6 scales. The ROC curves indicate sensitivity and specificity combined for all possible cutoff points, such that the area under the curve (AUC) is a measure of diagnostic accuracy (35). The interpretation of AUC values depends on the context: an AUC of 0.5 represents random recognition, an AUC of 0.9 is considered “excellent,” 0.8 is considered “good,” 0.7 is considered “fair,” and 0.6 is considered “poor” (36). Then, the optimal cutoff score shows the best trade-off between sensitivity and specificity. The highest Youden index (sensitivity + specificity - 1) was reported. The score corresponding to the highest Youden index was chosen as the cutoff.

## Results

### Step 1: Choose and Evaluate the Optimal Chinese Version of the STAI-S and STAI-T Short-Form Scales

#### Selection Process

We ranked the items according to their corrected item-total correlation coefficients to choose the most suitable items for the STAI-S scale, as shown in Table 2.

**TABLE 2:** Corrected item-total correlations of the STAI-S scale (*n* = 1142).

Name	Nature	*r*
STAI-15	P	**0.776**
STAI-18	N	**0.771**
STAI-2	P	**0.767**
STAI-10	P	**0.750**
STAI-17	N	**0.749**
STAI-19	P	**0.746**
STAI-8	P	**0.743**
STAI-5	P	0.729
STAI-6	N	**0.727**
STAI-4	N	**0.722**
STAI-16	P	0.720
STAI-13	N	**0.718**
STAI-7	N	0.716
STAI-1	P	0.715
STAI-20	P	0.715
STAI-9	N	0.706
STAI-3	N	0.704
STAI-11	P	0.697
STAI-12	N	0.641
STAI-14	N	0.544

*Correlations in bold indicate the five higher positively (P) and negatively (N) worded items.

Selecting half of the anxiety-present and half of the anxiety-absent items always yields the highest correlation coefficients. Based on the item-total correlation coefficient, four potential short scales, which contained 4, 6, 8, and 10 items, respectively, were generated. The 10-item scale (STAI-S-10) consisted of the top 5 anxiety-present and top 5 anxiety-absent items (anxiety-absent items: 15, 2, 10, 19, and 8; anxiety-present items: 18, 17, 6, 4, and 13). The 4-item scale (STAI-S-4), 6-item scale (STAI-S-6) and 8-item scale (STAI-S-8) were generated using the same approach.

Table 3 contains the Cronbach’s α coefficients of the four short-form scales and their correlation coefficients with the scores of the full-length STAI-S scale. The Cronbach’s α coefficients (>0.7) and correlation coefficients (>0.9) of these four short-form scales were acceptable (31, 32). Both the α coefficient and correlation coefficients increased with the addition of items.

**TABLE 3:** Cronbach’s α coefficients and correlation coefficients between the four short versions of the STAI-S scales and the full-length scale (*n* = 1,142).

Name	α	*r*
STAI-S-4 item	0.823	0.947
STAI-S-6 item	0.871	0.970
STAI-S-8 item	0.899	0.980
STAI-S-10 item	0.917	0.987

As shown in Table 4, the STAI-T scale was also ranked based on its corrected item-total correlation coefficients from highest to lowest. All scales contained the best-balanced anxiety-present and anxiety-absent items from the STAI-T scale and a higher corrected item-total correlation coefficient (anxiety-absent items: 33, 36, 30, 41, and 21; anxiety-present items: 38, 28, 31, 37, and 25).

**TABLE 4:** Corrected item-total correlations of the STAI-T scale (*n* = 1,142).

Name	Nature	*r*
STAI-33	P	**0.771**
STAI-36	P	**0.766**
STAI-30	P	**0.758**
STAI-38	N	**0.748**
STAI-41	P	**0.747**
STAI-21	P	**0.739**
STAI-23	P	0.731
STAI-28	N	**0.727**
STAI-31	N	**0.711**
STAI-37	N	**0.711**
STAI-25	N	**0.705**
STAI-39	P	0.703
STAI-32	N	0.701
STAI-27	P	0.696
STAI-42	N	0.688
STAI-22	N	0.684
STAI-26	P	0.683
STAI-29	N	0.683
STAI-35	N	0.683
STAI-40	N	0.665
STAI-43	N	0.643
STAI-44	N	0.617
STAI-34	P	0.585
STAI-24	P	−0.365

*Correlations in bold indicate the five higher positively (P) and negatively (N) worded items.

Table 5 describes Cronbach’s α coefficients for each of the four short-form scales and correlations between the four short-form scales with full-length STAI-T scales: 4-item scale (STAI-T-4), 6-item scale (STAI-T-6), 8-item scale (STAI-T-8) and 10-item scale (STAI-T-10). All comparisons had acceptable Cronbach’s α coefficients (>0.7 and >0.9) and correlation coefficients (31, 32).

**TABLE 5:** Cronbach’s α coefficients and correlation coefficients between the four short versions of the STAI-T scales and the full-length scale (*n* = 1,142).

Name	α	*r*
STAI-T-4 item	0.812	0.941
STAI-T-6 item	0.842	0.962
STAI-T-8 item	0.884	0.970
STAI-T-10 item	0.907	0.973

#### Evaluation Process

Although both the 4-item STAI-S and STAI-T scales had acceptable Cronbach’s α coefficients and correlations with the full-length scales, the construct validity was not ideal. For the STAI-S-4, the eigenvalue of the anxiety-present factor was 2.618 (65.4% of the variance) and that of the anxiety-absent factor was 0.736 (18.4% of the variance), which is less than 1. A similar result was obtained for the STAI-T-4 scale, the eigenvalue of the anxiety-present factor (2.564, 64.1% of the variance) and the anxiety-absent factor (0.807, 20.2% of the variance). Therefore, we evaluated the factor structure of the STAI-6 scale.

The KMO of the STAI-S-6 was 0.834 (X^2^ = 3636.285, *p* < 0.001), indicating that the adequacy of the model was high. A principal axis factor analysis with varimax rotation was used to evaluate the factor structure of the STAI-S-6 scale. Two factors were included in the analysis of eigenvalues and scree plots. The rotated factor loading data for this two-factor solution are described in Table 6. Two well-defined structures (anxiety-present and anxiety-absent) were identified. The anxiety-present factor was responsible for 60.90% of the variance, with an eigenvalue of 3.654. This factor contained three anxiety-present items and explained its structure. The anxiety-absent factor explained 17.25% of the variance, with an eigenvalue of 1.035. This factor was also consistent with three anxiety-absent items, which revealed its construct. All the items were significantly correlated with the sum of scores for the full scales.

**TABLE 6:** Principal axis factor analysis loading of the STAI-S-6 scale items following varimax rotation (*n* = 1,142).

State anxiety items			Factors	
	CR value	p	Anxiety-present	Anxiety-absent
10	31.093**	0.000	0.18	**0.89**
15	34.784**	0.000	0.27	**0.84**
2	36.993**	0.000	0.34	**0.78**
17	34.287**	0.000	**0.87**	0.25
18	37.084**	0.000	**0.85**	0.28
6	33.518**	0.000	**0.83**	0.25

**p < 0.001. The significant values are indicated in bold.

The KMO of STAI-T-6 was 0.832 (X^2^ = 3754.421, *p* < 0.001), indicating that the adequacy of the model was high. A principal axis factor analysis with varimax rotation was used to evaluate the factor structure of the STAI-T-6 scale. Again, an analysis of the eigenvalues and scree plot was carried out. Table 7 presents the rotated factor loading data for this two-factor solution. The anxiety-present factor was responsible for 60.47% of the variance, with an eigenvalue of 3.628. The anxiety-absent factor explained 18.27% of the variance, with an eigenvalue of 1.096. As shown in Table 7, the two well-defined structures were significantly separated into two factors, consistent with their interpretations. All the items were significantly correlated with the sum of scores for the full scales.

**TABLE 7:** Principal axis factor analysis loading of the STAI-T-6 scale items following varimax rotation (*n* = 1,142).

Trait anxiety items			Factors	
	CR value	p	Anxiety-present	Anxiety-absent
36	33.294**	0.000	**0.89**	0.23
30	34.216**	0.000	**0.88**	0.23
33	37.405**	0.000	**0.84**	0.30
31	31.339**	0.000	0.19	**0.85**
38	36.400**	0.000	0.27	**0.83**
28	31.157**	0.000	0.27	**0.81**

**p < 0.001. The significant values are indicated in bold.

**FIGURE 2:**
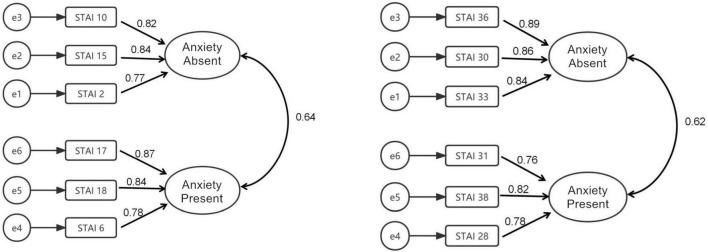
The confirmatory factor analysis model of STAI-S-6 and STAI-T-6.

AMOS 20. 0 was used to conduct confirmatory factor analysis (CFA) of the scale. The path coefficients were estimated and tested to evaluate and judge the fitting degree of the model according to the standard proposed in a previous study (37). The scale model and standardized path coefficient are shown in Figure 2. In terms of the parsimony fit index, confirmatory factor analysis is often used to test the similarity between the sample covariance matrix and the estimated covariance matrix through the ratio of chi-square to degrees of freedom (χ^2^/df). The indexes of the STAI-S-6 were χ^2^/df = 7.380, RMSEA = 0.075 (90% CI, 0.058–0.093), CFI = 0.986, NFI = 0.984, RFI = 0.970, IFI = 0.986, and TLI = 0.974; the indexes of the STAI-T-6 were χ^2^/df = 4.030, RMSEA = 0.052 (90% CI, 0.034–0.071), CFI = 0.994, NFI = 0.991, RFI = 0.984, IFI = 0.994, and TLI = 0.988 (see Table 8).

**TABLE 8:** Confirmatory factor analysis (CFA) of the short-form STAI scale (*n* = 1,142).

	χ ^2^/df	RMSEA	CFI	NFI	RFI	IFI	TLI
STAI-S-6	7.380	0.075	0.986	0.984	0.970	0.986	0.974
STAI-T-6	4.030	0.052	0.994	0.991	0.984	0.994	0.988

### Step 1: Test-Retest Reliability of the STAI-S-6 and STAI-T-6 Scales

Forty participants completed the STAI-S-6 and STAI-T-6 at a 1-week interval to determine their test-retest reliability. Thirty-five of the 40 respondents were female. The age range of the respondents was 18–25 years (*n* = 37), 26–30 years (*n* = 1), and 41–50 years (*n* = 2).

The average STAI-S-6 score was 11.88 ± 3.3 in the first test and 11.88 ± 3.49 in the second test. The average STAI-T-6 score was 12.33 ± 3.2 in the first test and 12.10 ± 3.29 in the second test. The rest-retest reliabilities of the STAI-S-6 and STAI-T-6 scales were investigated by calculating ICCs. The test-retest reliability of the STAI-S-6 scale was 0.728 (*p* < 0.001). The test-retest reliability of the STAI-T-6 scale was 0.813 (*p* < 0.001; see Table 9).

**TABLE 9:** Intraclass correlation coefficients (ICCs) for the scores of the STAI-S-6 and STAI-T-6 scales between the initial and second tests.

Variable	First test (*n* = 40) Mean ± SD	Second test (*n* = 40) Mean ± SD	ICC	*p*
STAI-S-6	11.88 ± 3.3	11.88 ± 3.49	0.728	<0.001
STAI-T-6	12.33 ± 3.2	12.10 ± 3.29	0.813	<0.001

### Step 2: Validity of the Short Chinese Versions of the STAI-S and STAI-6 and the Critical Receiver Operating Characteristic Value

#### Criterion Validity and Empirical Validity Compared With the Self-Rating Anxiety Scale and Satisfaction With Life Scale

Pearson’s correlation coefficient indicated a significant correlation between the STAI-S-6 and SAS scores (*r* = 0.289; *p* < 0.001), as shown in Table 10. The correlation coefficient between the STAI-S-6 and SWLS scores was −0.476 (*p* < 0.001). A similar correlation coefficient was observed for the STAI-T-6 score with the scores of the other scales (*r* = 0.313; *p* < 0.001 and *r* = −0.554; *p* < 0.001, respectively).

**TABLE 10:** Correlations of STAI-S-6 and STAI-T-6 scores with SAS and SWLS scores among participants without anxiety (*n* = 297).

	SAS (44.96 ± 8.40)		SWLS (21.18 ± 6.03)	
	*r*	*p*	*r*	*p*
STAI-S-6 (12.19 ± 3.53)	0.289	<0.001	−0.476	<0.001
STAI-T-6 (12.84 ± 3.36)	0.313	<0.001	−0.554	<0.001

Table 11 shows the means and standard deviations of the four scales. The independent sample *t* test showed that there was a significant difference between individuals without and with anxiety in terms of STAI-S6 and STAI-T-6 scores (*t* = −6.008; *p* < 0.001; Cohen’s *d* = 1.20; and *t* = −6.094; *p* < 0.001; Cohen’s *d* = 0.97). For the STAI-S-6 and STAI-T-6 scales, we also performed ANOVA to determine the difference between males and females among the individuals with and without anxiety. The variance of STAI-T-6 was not equal, and thus, we performed a log10 transformation of the data for homogeneity of variance. Significant differences were observed among the four groups of data; these differences showed that the STAI-S-6 and STAI-T-6 sufficiently distinguished the individuals without anxiety from individuals with anxiety (Levene’s statistic = 1.061, df1 = 3, df2 = 326, *p* = 0.366; *F* = 20.003; *p* < 0.001; η^2^ = 0.155, and Levene’s Statistic = 0.627, df1 = 3, df2 = 326, *p* = 0.598; *F* = 10.443; *P* < 0.001; η^2^ = 0.08, respectively). The LSD test was used to make multiple comparisons. Males (*p* = 0.019) and females (*p* < 0.001) with and without anxiety showed significant differences in STAI-S-6 scores. A significant difference in STAI-T-6 scores was not observed between males with and without anxiety (*p* = 0.197), but a significant difference in STAI-T-6 scores was noted between females with and without anxiety (*p* < 0.001).

**TABLE 11:** Empirical validity of the STAI-S-6 and STAI-T-6 in participants stratified by sex and mental state.

	No anxiety disorder (*n* = 297)		Anxiety disorder (*n* = 33)		Test index	*p*
STAI-S-6	12.19 ± 3.53		17.00 ± 4.44		*t*(36.63 df) = −6.008	<0.001
STAI-T-6	12.84 ± 3.36		16.76 ± 4.60		*t*(328 df) = −6.094	<0.001

	**Male (*n* = 116)**	**Female (*n* = 181)**	**Male (*n* = 12)**	**Female (*n* = 21)**		

STAI-S-6	12.59 ± 3.46	11.94 ± 3.57	15.17 ± 3.74	18.05 ± 4.56	F (3 df) = 20.003	<0.001
STAI-T-6	1.11 ± 0.11	1.08 ± 0.13	1.15 ± 0.11	1.24 ± 0.14	F (3 df) = 10.443	<0.001

#### Critical Receiver Operating Characteristic Value

**FIGURE 3:**
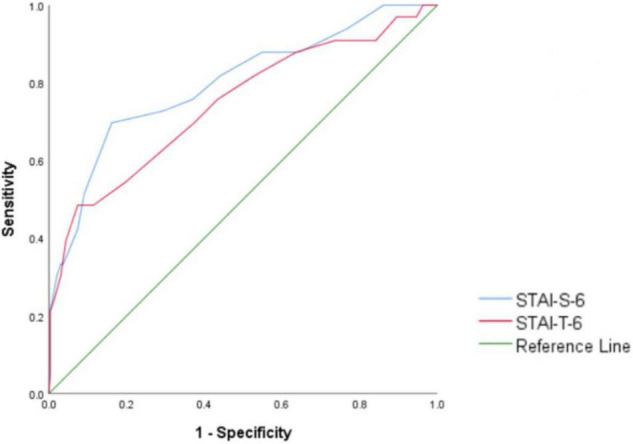
Receiver operating characteristic (ROC) curve of STAI-T-6.

As shown in Table 12, the accuracy of the STAI-S and STAI-T was fair for diagnosing major anxiety disorder, with AUCs of 0.798 and 0.745, indicating that the model displayed a good ability to predict the target variables. The effect was also observed in the ROC curve (Figure 3). The STAI-S-6 scale had the best screening performance, with a cutoff score > 15.5, a sensitivity of 69.7%, the highest Youden index of 0.535 and a specificity of 83.8%. According to the calculation of the sensitivity and specificity of STAI-T-6, the highest Youden index was 0.411, and the best cutoff point was greater than 17.5, indicating the best screening performance, with a sensitivity of 48.5% and a specificity of 92.6%.

**TABLE 12:** Area under the curve (AUC) analyses (*n* = 330).

				95% Confidence interval	
Index test	Area under the curve	Standard error	*P* value	Lower	Upper
STAI-S-6	0.798	0.045	<0.001	0.710	0.887
STAI-T-6	0.745	0.051	<0.001	0.645	0.845

## Discussion

Based on our research, the 6-item short forms of the STAI scales translated into Chinese showed excellent reliability and validity. The strong correlations between the original and abbreviated scales showed that the new versions are good alternatives to the longer original scales.

The aim of the present study was to produce short forms of the STAI scales, which are suitable for the situation in China, as studies have shown that the STAI is the most widely used tool for evaluating the state of anxiety (38, 39). However, fewer studies conducted in China have used the original STAI than studies abroad, perhaps due to the size of the scale.

In our study, Cronbach’s α was 0.871, and the total cumulative variance in the two factors was 78.15%. A previous study by Tian in China indicated that the Chinese version of the Short State Anxiety Inventory (SSAI) has acceptable indexes: Cronbach’s α = 0.81 (>0.7) (19), and the total cumulative variance in the two factors is 75.59%. We developed this Chinese version of the short scale of the STAI based on the Chinese sample and the full length of the Chinese STAI questionnaire. The correlation and structural validity of our short form of the STAI were improved by our findings. One of the other studies conducted in China that examined trait and state anxiety found that their scores differed in terms of sensitivity in representing the relationships of chronic mental health attributes and acute symptoms among Chinese secondary school students (40) using the Chinese version of the State-Trait Anxiety Inventory (C-STAI). We chose the best items, and thus, our statistical significance was higher, indicating that selecting items from the full length of the Chinese version of the STAI and then forming the new short form of the Chinese STAI scale would be more suitable for our current population than using a translated short form directly.

Anxiety is a stable disposition to respond to stress with anxiety and a tendency to perceive a wider range of situations as threatening. People with high trait anxiety according to the STAI-T have an attention bias toward danger information (41). The glycated hemoglobin level in patients with high trait anxiety is significantly higher than that in patients with low trait anxiety (42). Moreover, scores for the full version of the STAI-T are related to depression (43). The STAI-S was used to measure state anxiety, which is closely related to individual physical indicators such as respiratory rate, cardiac contraction and blood pressure (44). The short version of the STAI-S also performed well in respiratory patients (Cronbach’s α = 0.83, Pearson’s correlation coefficient *r* = 0.89 with the 20-item scale) (45). The ability to separate state and trait anxiety would help us investigate the correlation between anxiety status and mental or social status in greater depth. Even the GAD-7 scale reported by Shan and Li (46) has also been used in China to screen individuals who may have GAD, but significant differences between the anxious status that the GAD or STAI scales aim to distinguish have been noted. The two scales have different dimensions and applications. Generalized anxiety disorder is characterized by frequent or persistent, general, untargeted or fixed content of nervousness and excessive anxiety. The anxiety symptoms of individuals with GAD vary and consist of a range of physical and psychological symptoms. Compared to the GAD-7 scale, which provides a rapid assessment of generalized anxiety, the STAI is suitable for rapid assessment of various types of anxiety. The STAI aims to distinguish between state anxiety and trait anxiety. Trait anxiety refers to a relatively stable behavioral tendency of individuals to exhibit anxious responses to a wide range of threatening stimuli, while state anxiety is a transient mood state generated by the perception of dangerous stimuli. The STAI is suitable for adults with anxiety symptoms and has been widely used to assess anxiety in individuals related to internal medicine, surgery, psychosomatic disease and mental illness. It was also used to screen for related anxiety problems in college students, soldiers and other specific professional populations to evaluate the effectiveness of psychological therapy and drug therapy in a previous study (47).

We adopted SAS and SWLS as indicators to measure concurrent validity and found that the revised scale was positively correlated with the SAS score and negatively correlated with the SWLS score (*r* = −0.554, *P* < 0.001 and *r* = −0.476, *p* < 0.001), similar to the results of previous studies (48, 49). State anxiety is a transient emotional experience, and trait anxiety and life satisfaction are both related to the long-term evaluation of individuals; thus, the correlation coefficient between trait anxiety and life satisfaction may be higher. The results indicated the concurrent validity of the short STAI scale, and it can be used to study the correlation of mental health factors. We also supplemented the KMO and Bartlett’s test coefficients in the shortened STAI for structural validity. The KMO coefficient of the STAI-S-6 scale was 0.834, *P* < 0.001. The KMO coefficient of the STAI-T-6 scale was 0.832, *P* < 0.001. All of these values met the statistical requirements. The structural validity of the STAI was described in the evaluation process. The eigenvalues of the STAI-S-6 and STAI-T-6 scales in the two dimensions of the presence and absence of anxiety were both greater than 1, and the cumulative variance of the two scales was greater than 70% (78.15 and 78.74%). The research results fully support the good structural validity of the scale we developed. The correlation coefficients varied among previous studies. The specific relationship might be further investigated in subsequent basic research experiments, such as by assessing mediating effects or moderating effects. Regarding the correlation, the scales we developed show some clinical value for state and trait anxiety.

We used IRT to identify the items with the best discrimination parameters. Based on the original cutoff scores, we also proposed cutoff scores of 15.5 and 17.5 for the 6-item versions of the STAI-S and STAI-T, respectively. The moderate to strong correlations between the short forms of the scales and other measures evidenced sound external validity. Furthermore, a strong point of our study is that we not only tried to apply our short form of scale to the general population but also tried to compare the representativity of the effectiveness of the result of this short form with the result of the original scale.

The AUC of the STAI-S-6 scale was 0.798 and that of the STAI-T-6 scale was 0.745. According to the evaluation standard for the ROC curve mentioned in the methods, the STAI-S-6 scale was excellent at discerning individuals without anxiety from individuals with anxiety (AUC = 0.798), and the STAI-T-6 scale distinguished them fairly well (AUC = 0.745). From the results, the short version of the scale we developed clearly is better at diagnosing anxiety than the full scale (STAI-Y-2 AUC = 0.70) (6). However, the sensitivity of STAI-T-6 was much lower than 80%. This finding might be related to the characteristics of trait anxiety. It is part of the personality and not easily affected by the environment, and trait anxiety does not work responsively but initially influences other states, such as risk behavior, in relation to other mental factors. The participants may differ in personality types and levels of trait anxiety, potentially causing the results to have low sensitivity (50).

As we tried to prove the representativeness of the shout version of the scales compared with those reported in previous studies and the full version of the scales that previously been widely tested, this study presents a rare attempt to develop and validate a short-form version of the Chinese STAI. Based on the present results, both the STAI-S and STAI-T could be reduced to 6-item short-form scales without jeopardizing their psychometric properties. In the present study, the best items were carefully selected; multiple reliability and validity indexes of the STAI list were comprehensively evaluated in terms of retest reliability, internal consistency reliability, calibration validity, ROC curve characteristics, and construct-related validity; and their values were all within an acceptable range.

We compared the 4-, 6-, 8-, and 10-item STAI-S and STAI-T scales by comparing acceptable Cronbach’s α and correlation coefficients with the full-length scales. The construct-evaluated validity of the 4-item scales was not ideal. Since our goal of the study was to develop the shortest form of the STAI scale in Chinese, based on previous results, both the STAI-S and STAI-T can be reduced to 6-item short-form scales with acceptable validity and reliability (10). A translated version of the six-item short version in China was reported by Tian (22), but the authors expected that subsequent research would improve the limitations of their non-standard scale revision procedures. Our study has indicated that a better approach is to choose suitable items to form an effective short version of scales than merely translating scales that have been studied and summarized only abroad. The items included in the short version of the scale in this study were different from the items translated by Tian (22).

We also found that males were more likely than females to reach the cutoff. In Step 1, the ratio of anxiety in male participants to that in female participants completing the STAI-S-6 scale was 1.42, and the ratio of anxiety in male participants to that in female participants completing the STAI-T-6 scale was 1.36. In Step 2, the ratio of anxiety in male participants to that in female participants completing the STAI-S-6 scale was 1.43, and the ratio of anxiety in male participants to that in female participants completing the STAI-T-6 scale was 1.30. Previous reviews have suggested that females are indeed almost twice as likely to be affected by anxiety disorders than males (51). However, anxiety symptoms were not significantly different between sexes, but generally higher anxiety levels and prevalence scores were observed among the female groups. A potential explanation for these results might be that no single factor caused anxiety (52), but anxiety results from interactions among multiple risk factors, such as background stressors, financial status and family relationships, rendering females more susceptible to becoming anxious, but the difference is not highly significant. The findings of sex differences in anxiety symptoms in China are inconsistent with the research results from other countries. First, females were far less than twice as likely to experience anxiety as males. The prevalence of anxiety symptoms among female and male internal migrant workers in Shenzhen was 22.67 and 17.47%, respectively (the female-to-male ratio is 1.29) (53). Second, some studies reported no difference between men and women. The anxiety diagnosis rates in female and male middle school students were 31.78 and 26.06% (female-to-male ratio of 1.21), respectively. No significant differences in the distribution of anxiety symptoms were observed between sexes or grades (*P* > 0.05) (52). The age distribution of medical students in China is 18–30 years. The diagnosis rate of anxiety was 10.9%, and a significant difference was not observed between males and females (χ^2^ = 1.063, *P* = 0.303) (54). Among college students, a significant difference in anxiety scores was not observed between male and female students (*t* = 0.36, *P* = 0.717), and the positive detection rate was 23.9% (55).

The participants in this study primarily comprised young people aged between 18 and 30 years. At this stage, the diagnosis rate of males and females may be similar when they are faced with the same stressors. Subsequent studies should further explore whether a sex difference exists in anxiety among young and middle-aged people in developed cities and universities. In the current study, the selection of subjects, especially those with a clinical diagnosis, was not sufficient. Subsequent studies should increase the number of individuals who are diagnosed with anxiety to obtain a better ROC diagnostic threshold. The convergent validity of the STAI-S-6 and STAI-T-6 scales with the SAS was not high, and other anxiety instruments (e.g., the GAD scale) should be used. Depression-like SDS scales should be used to evaluate the divergent validity of the STAI-S-6 and STAI-T-6 scales in follow-up studies.

The correlations of scores for each item and the total STAI-T scale score were examined. In general, the internal consistency, reliability and validity of the scale might be affected by sampling. We found only one similar study that revised the STAI and assessed it in Chinese children (56); this study may have had the same purpose as our study. Future studies are needed to clarify not only the translation of the items but also to consider the modification of cultural background.

## Limitations

The ratio between individuals without anxiety and individuals with anxiety in our study was large (297 vs. 33). Although the AUC values performed well under disproportional conditions, the sensitivity of the STAI-T-6 was low (48.5%). A follow-up study should focus on the individuals who were diagnosed with anxiety to test whether the short Chinese version of the STAI would have better ROC values or sensitivity.

In follow-up studies, we recommend that researchers pay attention to the difference between trait anxiety and the relationship between trait anxiety and other mental or behavior states and possible mediating variables in this relationship.

## Conclusion

Six-item versions of the STAI scales, which are used as brief screening tools, provide an opportunity to identify people who may benefit from treatment and thus improve the mental health of the general population. We know that anxiety may cause somatization symptoms such as cardiovascular, digestive, respiratory, and urogenital autonomic dysfunction (57). Anxiety symptoms are also strongly correlated with insomnia symptoms (58, 59); trait anxiety is more frequently diagnosed in clinical patients than in the general population (58). Thus, early identification of anxiety is important for both the general and clinical populations. In particular, the influence of COVID-19 still interrupts regular mental health services, since we are unable to accurately predict outbreaks and people’s daily travels are restrained. This short-form survey may help both clinicians and people who want to quickly evaluate their mood status in the clinic or at home.

Since people’s attention span on the internet is shorter (60), the development of a short scale is necessary, but shorter scales are not necessarily better. The development of methods to make the scale as short as possible but still retain structural validity is very challenging and requires further research. Future studies should also consider modifying the items not only by considering the translation but also by understanding the items in the context of the cultural background of the population. Despite these shortcomings, this study has yielded short forms of one of the most widely used self-report measures of state and trait anxiety, and it also contributes to the evaluation and clinical application of the Chinese version of the STAI short scale.

## Data Availability Statement

The raw data supporting the conclusions of this article will be made available by the authors, without undue reservation.

## Ethics Statement

The studies involving human participants were reviewed and approved by Medical Ethics Committee of Southern Medical University. The patients/participants provided their written informed consent to participate in this study.

## Author Contributions

QD designed the study, wrote the protocol, and distributed the scale. HL and CY assisted with the scale distribution and managed the literature searches. QD and HL analyzed the data and wrote the first draft of the manuscript. QD, XC, and XZ edited the final manuscript. All authors contributed to, read and approved the final manuscript.

## Conflict of Interest

The authors declare that the research was conducted in the absence of any commercial or financial relationships that could be construed as a potential conflict of interest.

## Publisher’s Note

All claims expressed in this article are solely those of the authors and do not necessarily represent those of their affiliated organizations, or those of the publisher, the editors and the reviewers. Any product that may be evaluated in this article, or claim that may be made by its manufacturer, is not guaranteed or endorsed by the publisher.

## Abbreviations

STAI: State-Trait Anxiety Inventory
SAS: Self-Rating Anxiety Scale
SWLS: The Satisfaction with Life Scale
GAD: Generalized Anxiety Disorder
KMO: Kaiser–Meyer–Olkin: Measure of Sampling Adequacy.
